# A Mixed-Methods Cluster-Randomized Controlled Trial of a Hospital-Based Psychosocial Stimulation and Counseling Program for Caregivers and Children with Severe Acute Malnutrition

**DOI:** 10.1093/cdn/nzab100

**Published:** 2021-07-21

**Authors:** Allison I Daniel, Mike Bwanali, Josephine Chimoyo Tenthani, Melissa Gladstone, Wieger Voskuijl, Isabel Potani, Frank Ziwoya, Kate Chidzalo, Emmie Mbale, Anna Heath, Celine Bourdon, Jenala Njirammadzi, Meta van den Heuvel, Robert H J Bandsma

**Affiliations:** Centre for Global Child Health, Hospital for Sick Children, Toronto, Ontario, Canada; Translational Medicine Program, Hospital for Sick Children, Toronto, Ontario, Canada; Department of Nutritional Sciences, Temerty Faculty of Medicine, University of Toronto, Toronto, Ontario, Canada; The Childhood Acute Illness & Nutrition (CHAIN) Network, Blantyre, Malawi; The Childhood Acute Illness & Nutrition (CHAIN) Network, Blantyre, Malawi; Department of Women's and Children's Health, Institute of Translational Medicine, University of Liverpool, Liverpool, United Kingdom; The Childhood Acute Illness & Nutrition (CHAIN) Network, Blantyre, Malawi; Department of Pediatrics, College of Medicine, University of Malawi, Blantyre, Malawi; Amsterdam Centre for Global Child Health, Emma Children's Hospital, Amsterdam University Medical Centres, University of Amsterdam, Amsterdam, The Netherlands; Department of Nutritional Sciences, Temerty Faculty of Medicine, University of Toronto, Toronto, Ontario, Canada; The Childhood Acute Illness & Nutrition (CHAIN) Network, Blantyre, Malawi; The Childhood Acute Illness & Nutrition (CHAIN) Network, Blantyre, Malawi; The Childhood Acute Illness & Nutrition (CHAIN) Network, Blantyre, Malawi; The Childhood Acute Illness & Nutrition (CHAIN) Network, Blantyre, Malawi; Department of Pediatrics, College of Medicine, University of Malawi, Blantyre, Malawi; Child Health Evaluative Sciences, Hospital for Sick Children, Toronto, Ontario, Canada; Division of Biostatistics, Dalla Lana School of Public Health, University of Toronto, Toronto, Ontario, Canada; Department of Statistical Science, University College London, London, United Kingdom; Translational Medicine Program, Hospital for Sick Children, Toronto, Ontario, Canada; The Childhood Acute Illness & Nutrition (CHAIN) Network, Blantyre, Malawi; Department of Pediatrics, College of Medicine, University of Malawi, Blantyre, Malawi; Centre for Global Child Health, Hospital for Sick Children, Toronto, Ontario, Canada; The Childhood Acute Illness & Nutrition (CHAIN) Network, Blantyre, Malawi; Division of Pediatric Medicine, Hospital for Sick Children, Toronto, Ontario, Canada; Department of Pediatrics, Temerty Faculty of Medicine, University of Toronto, Toronto, Ontario, Canada; Centre for Global Child Health, Hospital for Sick Children, Toronto, Ontario, Canada; Translational Medicine Program, Hospital for Sick Children, Toronto, Ontario, Canada; Department of Nutritional Sciences, Temerty Faculty of Medicine, University of Toronto, Toronto, Ontario, Canada; The Childhood Acute Illness & Nutrition (CHAIN) Network, Blantyre, Malawi; Department of Pediatrics, Temerty Faculty of Medicine, University of Toronto, Toronto, Ontario, Canada; Department of Biomedical Sciences, College of Medicine, University of Malawi, Blantyre, Malawi

**Keywords:** severe acute malnutrition, SAM, child development, nutrition, HOME Inventory, psychosocial stimulation, nurturing care framework

## Abstract

**Background:**

Children with severe acute malnutrition (SAM) who require nutritional rehabilitation unit (NRU) treatment often have poor developmental and nutritional outcomes following discharge. The Kusamala Program is a 4-d hospital-based counseling program for caregivers of children with SAM that integrates nutrition, water, sanitation, and hygiene and psychosocial stimulation, aimed at improving these outcomes.

**Objectives:**

The aim was to evaluate the effects of the Kusamala Program on child development and nutritional status in children with SAM 6 mo after NRU discharge. The other aim was to qualitatively understand perceptions and experiences of caregivers who participated in the intervention.

**Methods:**

A cluster-randomized controlled trial was conducted with caregivers and their children 6–59 mo of age with SAM admitted to the Moyo NRU in Blantyre, Malawi. The primary outcome of the trial was child development according to Malawi Developmental Assessment Tool (MDAT) composite *z-*scores of gross motor, fine motor, language, and social domains. A qualitative component with focus group discussions and in-depth interviews was also completed with a subset of caregivers who participated in the trial.

**Results:**

Sixty-eight caregivers and children were enrolled to clusters by week and randomly assigned to the comparison arm and 104 to the intervention arm. There were no differences in child development, with mean MDAT composite *z*-scores in the comparison arm of −1.2 (95% CI: −2.1, −0.22) and in the intervention arm of −1.1 (95% CI: −1.9, −0.40) (*P* = 0.93). The qualitative evaluation with 20 caregivers indicated that the 3 modules of the Kusamala Program were appropriate and that they applied many of the lessons learned at home as much as possible.

**Conclusions:**

The Kusamala Program did not result in improved developmental or nutritional outcomes, yet it was viewed positively by caregivers according to qualitative results. Future research should evaluate more intensive interventions for caregivers and children with SAM. This trial was registered at www.clinicaltrials.gov as NCT03072433.

## Introduction

Children who have experienced severe acute malnutrition (SAM), which presents as severe wasting or as edematous malnutrition, are at high risk of poor nutritional outcomes like impaired linear growth as well as inadequate development across multiple domains (1–6). Recent data showed that surviving children who were assessed 7 y after having SAM were more likely to be at an earlier grade in school and have lower cognitive test scores compared with peers who did not have SAM (7).

Because of the association between malnutrition and negative developmental outcomes, the 2003 WHO guideline for inpatient treatment of children with SAM recommends psychosocial stimulation interventions at nutritional rehabilitation units (NRUs) (8). Furthermore, in addition to psychosocial stimulation interventions, nutritional counseling was recommended as part of the guideline (8). There are also potential links between water, sanitation, and hygiene (WASH) and outcomes including child development in this population, particularly because enteric dysfunction is common in children with SAM and especially in those who have an acute illness (9–12).

However, the underlying evidence for these recommendations is limited, with few studies evaluating psychosocial stimulation programs for these children (8, 13–16). Although this past research showed that psychosocial stimulation interventions improved developmental outcomes, they were based on very intensive programs that began during the NRU admission period and continued in community or home settings (14–16). It is still unknown whether psychosocial stimulation and possible other interventions to target child development are feasible in NRU settings in practice and what intensity of inpatient and postdischarge support might be needed to achieve change. Importantly, previous studies also included children with SAM without clinical complications, whereas children admitted to NRUs now based on the current guidelines have complications such as HIV infection, diarrhea, or severe edema (6, 17, 18).

It is clear that present-day implementation research is needed to understand how to improve child development in children with SAM who require inpatient treatment in a feasible and pragmatic way. Our team therefore designed the Kusamala Program, a counseling intervention led by nurses in an NRU setting involving primary caregivers of children with SAM (19). We aimed to create a focused program that could be delivered beginning within 3 d of admission to the NRU and completed before the time of discharge. The Kusamala Program incorporated 3 different modules, including nutrition and feeding, WASH, and psychosocial stimulation, with supervised play sessions, which are described in more detail in the Methods section and in previous publications (19, 20). We hypothesized that psychosocial stimulation interventions combined with additional nutritional counseling and WASH modules would improve developmental and nutritional outcomes in children with SAM in an NRU setting. We previously undertook a feasibility study to understand the potential for implementation of the Kusamala Program in this setting delivered by nurses working in this ward. The first aim of the feasibility study was to assess participant engagement and adherence based on attendance to assess whether caregivers were willing to attend sessions in this environment. Another aim was to qualitatively identify barriers and enablers to its implementation in an NRU setting, which led to modifications of the intervention such as shortening sessions (20).

The primary objective of this research was to evaluate the effectiveness of the Kusamala Program to improve developmental outcomes based on Malawi Developmental Assessment Tool (MDAT) *z*-scores and nutritional status in children with SAM 6 mo after NRU discharge (21). An additional objective was to assess the impact of the program on care practices in the home setting following inpatient treatment. In addition to these quantitative evaluations, we aimed to qualitatively understand the perspectives of primary caregivers who participated in the Kusamala Program. We embedded this qualitative research within the cluster-randomized controlled trial for interpretation of the quantitative results, including care practices and child outcomes. These qualitative data were also important for understanding implementation and appropriateness of the program from a participant perspective to augment our previous feasibility assessments and implementation research within this study (20).

## Methods

### Cluster-randomized controlled trial design

Between 1 and 6 caregivers and their children were recruited by 1 of 2 field workers weekly into clusters. Each cluster was randomly assigned to the intervention or comparison arm between November 2016 and May 2020. The reason for the cluster design was to prevent overlap between intervention and comparison weeks. Potential participants were screened for eligibility within 3 d after admission to the Moyo Nutritional Rehabilitation and Research Unit (Moyo NRU) at Queen Elizabeth Central Hospital (University of Malawi College of Medicine) in Blantyre, Malawi, by trained field workers who were blinded to the allocation, as informed consent was sought before randomization. Randomization was based on an allocation scheme created by a statistician, with the allocation in sealed envelopes that were opened after recruitment. Further details of this process have been published in the protocol paper (19).

Caregivers and children in the intervention arm of the trial were provided with the Kusamala Program. Those in the comparison arm were given the current standard of care, which included counseling by nurses on basic nutrition and WASH messages at the time of discharge. All children were given standard nutritional and clinical care, consistent with WHO and national guidelines, beginning with a stabilization phase of treatment followed by a rehabilitation phase (18). The inclusion criteria were as follows: *1*) child between 6 and 59 mo with SAM [weight-for-height *z*-score (WHZ) below −3 SDs, midupper arm circumference (MUAC) <115 mm, and/or nutritional edema] admitted to an NRU due to the presence of medical complications, severe oedema, or loss of appetite, and *2*) primary caregiver (self-identified) present at the hospital. The exclusion criteria were as follows: *1*) primary caregiver declined to give informed consent and *2*) child with a known terminal illness likely to cause death within 6 mo according to the treating physician or the child requires a surgical procedure. Children with neuro-disabilities such as cerebral palsy, based on identification by a clinician, were eligible to be enrolled in the trial yet omitted from the main statistical analysis.

### The Kusamala Program

This intervention was a 4-d interactive counseling program for primary caregivers of children with SAM receiving treatment at the Moyo NRU. It involved 3 different modules: nutrition and feeding, WASH, and psychosocial stimulation. One module was covered each day followed by a summary session on the final day. Participants were each given take-home images for each of the nutrition and WASH sessions, a toy for the psychosocial stimulation session, and a certificate at the end of completion of the Kusamala Program. The intervention was led by 5 nurses working at the NRU. The duration of each session was set to be 75 min, including time for counseling and interactive play activities, based on results from the feasibility study including nurses and other NRU staff involved in the Kusamala Program (20). Further methods about the design and practice of the Kusamala Program are described in the protocol paper (19).

Attendance was documented at each of the Kusamala Program sessions by the nurses who delivered the program that particular week. Intervention fidelity was assessed by a Malawian investigator with strong expertise in child development and psychosocial stimulation (JCT). This involved observing sessions for every fifth Kusamala Program cluster. The assessment for each session included the duration of the intervention; if nurses covered key messages (3 for nutrition and feeding and for WASH sessions; 2 for the psychosocial stimulation session); whether the corresponding take-home image, toy, or certificate was given at each session; the overall quality of delivery based on a 5-point Likert scale; and an evaluation of 10 counseling skills from the Care for Child Development manual (22). Refresher training for nurses delivering the Kusamala Program was provided twice per year, with feedback from the fidelity assessments used to supplement this training.

### Quantitative data collection

Quantitative data were collected at enrollment, at discharge from the NRU, and 6 mo after discharge at the homes of caregivers and children. These assessments were done by trained field workers (FZ, KC, and JCT) who were blinded to the allocation. The quantitative data collected were de-identified and entered into a password-secure Research Electronic Data Capture database shortly after data collection, which was only accessible by 2 study investigators (AID and MB).

The primary outcome measure was the MDAT, a context-appropriate tool for assessing development. It has been validated in a reference population of Malawian children and validated against age-matched children with malnutrition and those with neuro-disabilities (21). The MDAT involves an enumerator assessing children's abilities to complete tasks of increasing difficulty, up to 36 items for each of gross motor, fine motor, language, and social domains. It has good inter- and intraobserver reliability, with nearly all items having Cohen's κ values >0.4 (21). We conducted an MDAT assessment at discharge from the hospital and 6 mo after discharge, yet we did not complete the MDAT at enrollment children as we considered that many children may be too sick for assessments until they have been clinically stabilized, which is the first phase of inpatient treatment (18).

Other key outcomes, which were assessed in duplicate at all time points, including enrollment, discharge, and 6 mo after discharge, were child nutritional status based on anthropometric measures, including bilateral pitting edema, absolute MUAC, and WHZ, height-for-age *z*-score (HAZ), and weight-for-age *z*-score (WAZ) calculated according to the 2006 WHO Child Growth Standards (23). Additional data collected at the 6-mo follow-up included readmission to the hospital for SAM treatment and mortality; care practices, including 24-h dietary recall; and the care environment using the Home Observation of the Measurement of the Environment (HOME) Inventory (24).

### Sample size and futility analysis

As there were no prior data on MDAT *z*-scores in children with SAM following NRU discharge, we estimated the sample size using data from an internal pilot study within our trial (20). We estimated 320 children across the 2 arms were needed to detect a medium effect size of 0.5 using Cohen's *d* between trial arms, increased to 400 children to account for contingencies.

Over the course of the trial, there were substantial difficulties with recruitment, predominantly due to a steady decline in NRU admissions, from 1438 children per year at the Moyo NRU in 2009 to 337 in 2019. A decision was made to examine the scientific futility of the trial, which is when there is no evidence of a benefit of the intervention (25–27). The aim was to calculate the probability of an effect, based on the data already collected within the study, and to use this to decide on continuing or stopping the trial.

The futility analysis methods were adapted from published methods by a statistician involved in this project (AH) (28). The futility analysis was undertaken in R 3.6.0 (29). All available interim data collected by May 2020, 3.5 y after the cluster-randomized controlled trial's start date, were used for this analysis. In alignment with the trial methods, the MDAT composite *z*-score at follow-up was used as the primary outcome. The power for the proposed futility analysis, based on predictive power, was assessed using simulations with an effect size of 0.5 using Cohen's *d* (30). A predictive power cutoff of 20%, a commonly used threshold (31), was agreed upon by study investigators before completing the futility analysis. This meant that if the predictive probability of a significant result at the end of the trial was <20% then the trial would be stopped for futility. The trial sample size was increased to 328 across the 2 arms (adjusted to 410) to maintain 80% power.

### Statistical analysis of quantitative data

Data were exported from the Research Electronic Data Capture database prior to analysis using Stata 16 (StataCorp LP) (32, 33). Means (95% CIs) were calculated for continuous variables and percentages for categorical variables.

We created age-adjusted MDAT composite *z*-scores, consolidated from items from all 4 domains, as well as MDAT *z*-scores for each individual domain, based on data from a reference population of children in Malawi using the MDAT Scoring Application version 1.1 (21, 34). We calculated anthropometric *z*-scores using the *zscore06* macro in Stata 16 (23, 35). MDAT and anthropometric *z*-scores were considered implausible if they were below −6 or >6 SD for HAZ and MDAT *z*-scores, and up to 5 SDs for WHZs and WAZs (23).

We completed multiple imputation by chained equations for children with missing data who were not confirmed to have died during the study. This is a robust technique for data that are missing at random or missing completely at random. It uses a separate probability distribution for each imputed variable. Child HIV status, sex, and baseline weight, height, and edema were included as variables in the imputation model. Twenty imputed datasets were created for each of the main outcomes of interest, including MDAT composite *z*-scores, MDAT *z*-scores for each individual domain, anthropometric indices, HOME Inventory scores, and dietary diversity scores. Multiple imputation results were compared with available case analysis to assess the influence that missing data had on the results.

We analyzed data with intention-to-treat at the participant level with multiple linear regression to determine differences in outcomes across arms. Covariates included in the model were child variables including HIV status, age, and sex; caregiver variables including educational level, nutritional status, HIV status, age, and marital status; household income in the previous month; and urban or rural household location. Clustered robust SEs were used for regression.

### Qualitative data collection

Caregivers who participated in the trial were approached to participate in the qualitative study if their follow-up data collection visit was completed so as to not unblind study personnel before participants finished the study. Convenience sampling was undertaken by selecting participants who were reachable by phone. In total, 25 caregivers were contacted to participate in the qualitative study and 5 did not attend for unknown reasons. A sample size of 20 was considered to be acceptable and was assessed during the qualitative study to confirm this was sufficient to reach data saturation (36).

In-depth interviews (IDIs) and focus group discussions (FGDs) were done by the same investigator who was trained in qualitative methods including thematic analysis (JCT). The investigator had experience with the cluster-randomized controlled trial including quantitative data collection and fidelity assessments of the Kusamala Program. Some participants in the qualitative study were therefore familiar with the investigator at the time of the IDIs and FGDs. There were no interviewer characteristics that were likely to influence the qualitative study but did have specific interest in this research topic. At the time of the qualitative study, the investigator did not have knowledge of the results of the cluster-randomized controlled trial of the effectiveness of the Kusamala Program.

Data collection was completed at Queen Elizabeth Central Hospital in a private office setting with only the participants and investigator present. The interviews were conducted in Chichewa, the primary local language in Malawi, using a semi-structured topic guide that was translated from English to Chichewa. Initial questions were more closed, with the investigator asking probing questions throughout to deepen the dialogue within the IDIs and FGDs. The main questions included the following:

1. What do you recall from the Kusamala Program? Did you have positive or negative experiences, and how comfortable were you in this learning environment?
2. What were the goals of the Kusamala Program? Did the intervention change your knowledge in any way?
3. Do you have enough resources at home to carry out what you learned in the Kusamala Program? Are there any other barriers to implementing what was taught in the intervention?
4. Were there any topics that were not important as part of the Kusamala Program? Were there additional topics that you would have been interested in learning about?
5. Have you shared anything you learned with other people that you know in the community? Are there other settings or participants who could benefit from this type of program?

IDIs and FGDs were recorded using an audio device and the investigator took notes throughout. The duration ranged from 45 to 90 min. Following completion of the interviews and discussions, audio recordings were transcribed and then translated to English by study personnel fluent in English and Chichewa. Throughout these processes, the investigator conducting the qualitative study considered whether data saturation was achieved, which was the case with 20 participants.

### Qualitative analysis and themes

The main questions to answer and corresponding themes were identified in advance, although subthemes were derived from the data. Qualitative content analysis was done to understand these 3 main themes: how participants felt about the content of the Kusamala Program; its conduct in an NRU, as well as other settings or children and caregivers who could benefit; and whether caregivers were able to apply practices from the intervention at home. The content analysis was also completed to gauge the sentiment of caregivers towards the Kusamala Program, whether positive or negative, by evaluating detailed descriptions of participants about why the intervention was or was not valuable to them.

Data were analyzed by 2 different authors (JCT and AID) including the investigator who carried out the qualitative study. Coding was done in NVivo 12 (QSR International) independently using inductive coding within the 3 main themes that were pre-planned for the content analysis and discussed in detail following this process to achieve consensus (37). Caregivers did not provide feedback on the findings because of the difficulty in tracing participants, yet the investigator summarized what was described by participants at the end of each IDI and FGD to ensure an understanding of the key messages.

### Reporting guidelines

The CONSORT (Consolidated Standards of Reporting Trials) 2010 statement: extension to cluster randomized trials was followed for the reporting of this research (38). As stated previously, more detailed methods are described in the protocol publication for this cluster-randomized controlled trial (19). For the qualitative study, the COREQ (Consolidated Criteria for Reporting Qualitative Research) checklist was followed (39).

### Ethical approval

Ethical preapproval for the cluster-randomized controlled trial and the qualitative component of the study was obtained from the University of Malawi College of Medicine Research and Ethics Committee (P.05/16/1930) and the Hospital for Sick Children Research Ethics Board (1,000,053,578). The trial was registered at ClinicalTrials.gov (NCT03072433) in March 2017 after beginning to enroll participants to the internal pilot trial in November 2016, which was previously published elsewhere (20).

## Results

**FIGURE 1:**
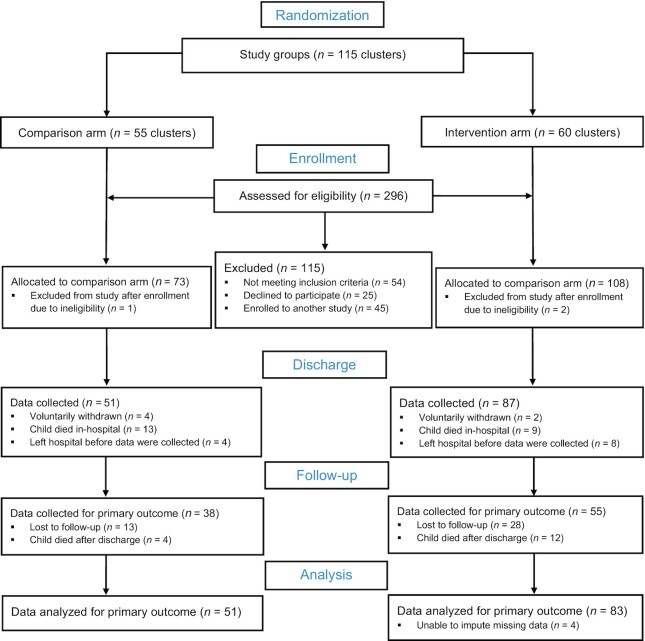
Study flow chart for the cluster-randomized controlled trial.

Recruitment for the trial started in November 2016 and continued until May 2020, with 296 children admitted to the Moyo NRU assessed for eligibility within this time frame (**Figure 1**). There were 115 clusters across the 2 study arms, with 73 children and their caregivers allocated to 55 clusters in the comparison arm and 108 children and their caregivers allocated to 60 clusters in the intervention arm based on the randomization scheme.

The attendance for participants in the intervention group, including only children who survived hospital stay, was 93.1% on day 1, 89.1% on day 2, 79.2% on day 3, and 64.4% on day 4 of the Kusamala Program. Fidelity assessments were done for 11 Kusamala Program weeks, which covered 36 different sessions between October 2017 and March 2020. Eight Kusamala Program sessions within these 11 wk were not completed because participants were discharged before the end of the 4 d of the intervention. The mean duration of each session was 65.9 (95% CI: 60.0, 71.9) min. 97.8% (89/91) of key messages were covered in these sessions, and 94.4% (34/36) of the corresponding take-home images, toys, and certificates were given to participants. The average overall quality rating based on the Likert scale was 4.25 (95% CI: 4.04, 4.46) out of 5. 89.7% (323/360) of counseling skills were met across sessions from the Care for Child Development manual (22).

### Futility analysis to stop the trial

Follow-up continued until May 2020 after the onset of the coronavirus disease 2019 (COVID-19) pandemic in Malawi, coinciding with when investigators planned to conduct the futility analysis after difficulties in recruitment across the first 3.5 y of the trial due to a major decline in NRU admissions. The futility analysis showed that the probability of a significant result in the MDAT composite *z*-score as the primary outcome was 18%, which was below the threshold of 20%. Because of this result, recruitment for the trial was stopped for futility in May 2020. The COVID-19 pandemic made it unsafe for field workers to conduct additional follow-ups after this period, so follow-up data were not collected beyond when the futility analysis was completed. In total, 13 of 55 (23.6%) children and caregivers were lost to follow-up in the comparison arm and 28 of 95 (29.5%) in the intervention arm over the course of the study.

### Quantitative results of the cluster-randomized controlled trial

Child, caregiver, and household characteristics at baseline were similar across the 2 study arms (**Table 1**). The mean duration of stay in the NRU was 7.8 (95% CI: 5.9, 9.7) d for children in the comparison arm and 8.7 (95% CI: 7.4, 9.9) d for children in the intervention arm (*P* = 0.41). Inpatient mortality in children admitted to the Moyo NRU was higher in the comparison arm, at 13/68 (19.1%), contrasted with 9/104 (8.7%) in the intervention arm (*P* = 0.045). This is unlikely related to the Kusamala Program, as many NRU deaths occur within the first days upon hospital admission, at which point the intervention would not yet be completed. In children with neuro-disability, inpatient mortality rates were 2/18 (11.1%) in the comparison arm and 1/12 (8.3%) in the intervention arm (*P* = 0.80). The mean duration of stay was 8.3 (95% CI: 4.2, 12.3) d for children with neuro-disability in the comparison arm and 11.2 (95% CI: 5.5, 16.8) d in the intervention arm.

**FIGURE 2:**
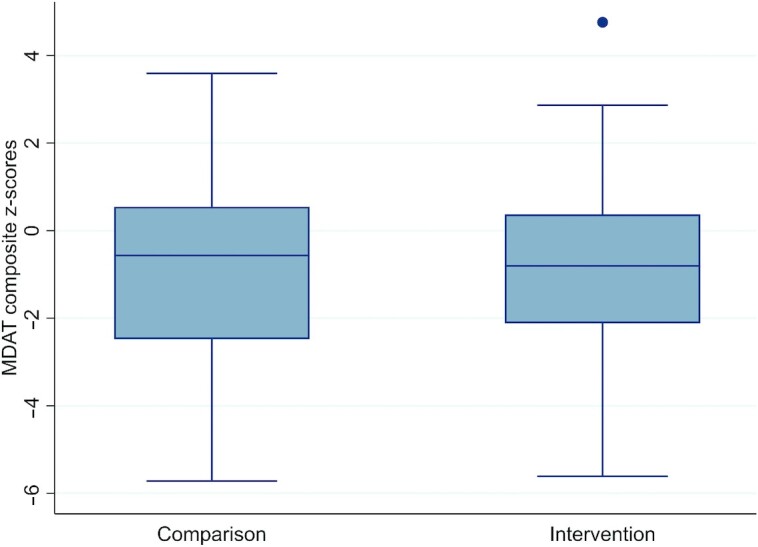
Developmental outcomes according to MDAT composite *z*-scores in children 6 mo after discharge from inpatient treatment of severe acute malnutrition between the comparison and intervention arms. The point above the intervention boxplot represents an outlier. MDAT, Malawi Developmental Assessment Tool.

**FIGURE 3:**
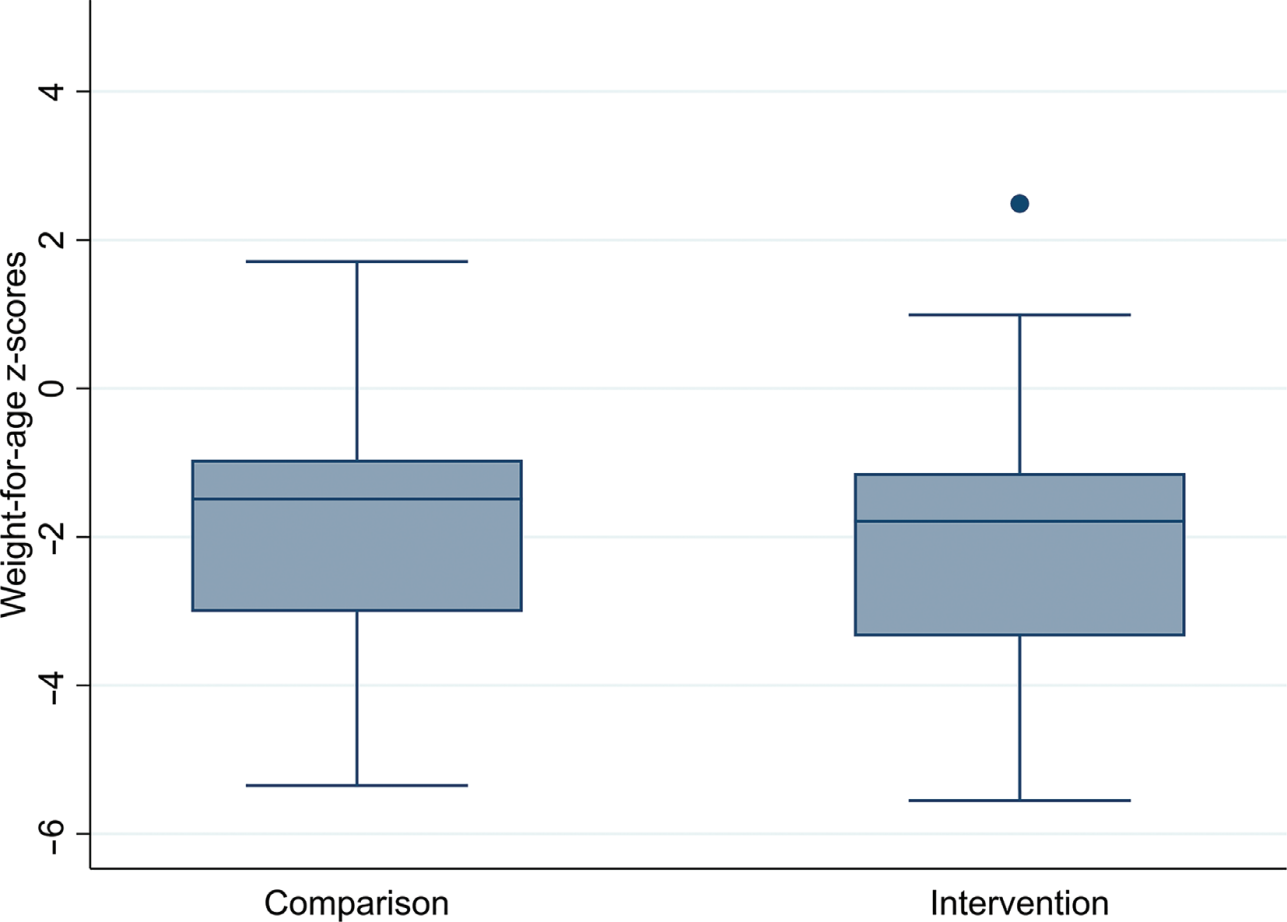
Nutritional status according to weight-for-age *z*-scores in children 6 mo after discharge from inpatient treatment of severe acute malnutrition between comparison and intervention arms. The point above the intervention boxplot represents an outlier.

**TABLE 1:** Baseline characteristics of children admitted for inpatient treatment of severe acute malnutrition and their caregivers and households between comparison and intervention arms^1^

	Comparison (*n* = 68)	Intervention (*n* = 104)
Age, mo	22.1 ± 12.9	19.1 ± 10.1
Sex, female, *n*/total *n* (%)	32/68 (47.1)	48/104 (46.1)
HIV positive, *n*/total *n* (%)	17/68 (25.0)	33/102 (32.4)
Edema, *n*/total *n* (%)	28/68 (41.2)	36/103 (35.0)
MUAC,^2^ cm	11.0 ± 1.0	10.8 ± 1.1
WHZ^2^	−3.3 ± 1.1	−3.4 ± 1.0
HAZ	−3.0 ± 1.7	−2.9 ± 1.9
WAZ^2^	−3.9 ± 1.1	−4.0 ± 1.5
Caregiver relationship to child, *n*/*n* (%)		
Mother	59/67 (88.1)	101/104 (97.1)
Other	8/67 (11.9)	3/104 (2.9)
Caregiver age, y	26.8 ± 8.2	27.3 ± 6.7
Caregiver BMI, kg/m^2^	22.9 ± 4.7	21.9 ± 2.6
Caregiver depressive symptoms (Self-Reporting Questionnaire 20), median (IQR)	4 (8)	3 (6)
Monthly household income, USD equivalent	22.96 ± 26.41	29.59 ± 34.42
Number of children in the household	3.0 ± 1.4	2.9 ± 1.5
Household area, *n*/total *n* (%)		
Urban	41/67 (61.2)	62/104 (59.6)
Rural	26/67 (38.8)	42/104 (40.4)

1 Values are means ± SDs unless otherwise indicated. HAZ, height-for-age *z*-score; MUAC, midupper arm circumference; USD, US dollars; WAZ, weight-for-age *z*-score; WHZ, weight-for-height *z*-score.

2 Excluding children with edema.

Primary outcome data were collected for 38 children in the comparison arm and 55 in the intervention arm at follow-up 6 mo after discharge (Figure 1). In the comparison arm, there were 13 children lost to follow-up and 4 children who died after discharge; in the intervention arm there were 28 lost to follow-up and 12 who died after discharge. Values were imputed for these children who were lost to follow-up. In addition to these missing data, there were 3 MDAT composite *z*-score values out of range for the comparison arm and 2 for the intervention arm, so these were treated as missing data and were imputed. Furthermore, there was 1 value out of range for the MDAT gross motor domain, 4 out of range for the fine motor domain, 2 out of range for the language domain, and 5 out of range for the social domain.

There were no differences between arms in terms of MDAT composite *z*-scores or individual domains based on the available case analysis and multiple imputation results (**Table 2** and **Figure 2**). There were also no differences in nutritional status based on MUAC, WHZ, HAZ, and WAZ across the 2 arms (Table 2 and **Figure 3**). Nutritional outcomes also did not differ between arms for children with neuro-disability. In these children, mean MUAC was 13.6 cm (95% CI: 12.6, 14.6 cm) for those in the comparison arm and 12.4 cm (95% CI: 10.9, 13.9 cm) in the intervention arm (*P* = 0.12). For WAZ, the mean was −3.4 (95% CI: −5.1, −1.8) for children with neuro-disability in the comparison arm and −3.8 (95% CI: −5.2, −2.4) in the intervention arm (*P* = 0.68).

**TABLE 2:** Developmental and nutritional outcomes in children 6 mo after discharge from inpatient treatment of severe acute malnutrition between the comparison and intervention arms^1^

	Available case analysis			Multiple imputation		
	Comparison (*n* = 35)	Intervention (*n* = 53)	*P*	Comparison (*n* = 51)	Intervention (*n* = 83)	*P*
MDAT *z*-score						
Composite	−0.82 (−1.6, −0.070)	−0.93 (−1.5, −0.35)	0.80	−1.2 (−2.1, −0.22)	−1.1 (−1.9, −0.40)	0.93
Gross motor	−0.53 (−1.1, 0.049)	−0.76 (−1.3, −0.27)	0.53	−0.57 (−1.2, 0.086)	−0.75 (−1.4, −0.15)	0.55
Fine motor	−0.86 (−1.6, −0.14)	−0.33 (−0.89, 0.24)	0.46	−1.0 (−1.9, −0.15)	−0.44 (−1.2, 0.29)	0.48
Language	−0.45 (−1.1, 0.23)	−0.66 (−1.1, −0.18)	0.57	−0.47 (−1.4, 0.43)	−0.66 (−1.2, −0.11)	0.51
Social	−0.67 (−1.2, −0.11)	−0.88 (−1.3, −0.45)	0.57	−0.74 (−1.5, −0.033)	−0.99 (−1.5, −0.45)	0.43
MUAC,^2^ cm	13.9 (13.3, 14.5)	13.8 (13.4, 14.2)	0.67	13.8 (13.1, 14.4)	13.8 (13.2, 14.4)	0.87
WHZ^2^	−0.27 (−1.0, 0.48)	−0.40 (−0.89, 0.097)	0.69	−0.41 (−1.3, 0.45)	−0.24 (−0.93, −0.45)	0.86
HAZ	−3.4 (−3.9, −3.8)	−3.2 (−3.7, −2.7)	0.48	−3.4 (−4.2, −2.7)	−3.4 (−4.1, −2.7)	0.73
WAZ^2^	−1.9 (−2.5, −1.3)	−2.1 (−2.5, −1.7)	0.81	−2.2 (−2.8, −1.5)	−2.0 (−2.5, −1.5)	0.88

1 Values are means (95% CIs). Models adjusted for child HIV status, age, and sex; caregiver educational level, nutritional status, HIV status, age, and marital status; and household income and urban or rural location.

HAZ, height-for-age *z*-score; MDAT, Malawi Developmental Assessment Tool; MUAC, midupper arm circumference; WAZ, weight-for-age *z*-score; WHZ, weight-for-height *z*-score.

2 Excluding one child with oedema in the comparison arm.

With regard to other child outcomes, posthospital discharge mortality reported by caregivers who were successfully followed up was 4/42 (10.5%) for the comparison arm and 12/67 (17.9%) in children who were allocated to the Kusamala Program (*P* = 0.31). Readmission to an NRU in children who survived inpatient treatment was 7/36 (19.4%) for children in the comparison arm and 10/57 (17.5%) in the intervention arm (*P* = 0.82). The postdischarge mortality rate in children with neuro-disability was 2/14 (14.3%) in the comparison arm and 3/11 (27.3%) in the intervention arm (*P* = 0.45).

Care practices including HOME Inventory scores and 24-h dietary recall scores were similar between arms at follow-up, indicating that the Kusamala Program did not change these caregiving behaviors (**Table 3**).

**TABLE 3:** Care practices of children 6 mo after discharge from inpatient treatment of severe acute malnutrition between the comparison and intervention arms^1^

	Available case analysis			Multiple imputation		
	Comparison (*n* = 38)	Intervention (*n* = 55)	*P*	Comparison (*n* = 51)	Intervention (*n* = 83)	*P*
HOME Inventory scores (out of 25)	16.9 (16.0, 17.9)	17.2 (16.2, 18.2)	0.54	16.9 (15.5, 18.2)	16.9 (15.8, 18.1)	0.70
Dietary diversity scores (out of 7)	3.6 (3.1, 4.1)	3.6 (3.2, 3.9)	0.42	3.6 (3.0, 4.2)	3.6 (3.1, 4.0)	0.42

1 Values are means (95% CIs). Models adjusted for child HIV status, age, and sex; caregiver educational level, nutritional status, HIV status, age, and marital status; and household income and urban or rural location.

HOME, Home Observation for Measurement of the Environment.

### Qualitative evaluation of the Kusamala Program

A total of 7 IDIs and 4 FGDs were conducted with 20 participants between 9 January 2020 and 2 April 2020. Four of these caregivers were mothers of children who died following discharge from the NRU.

#### Kusamala Program modules: “I can still be talking to my child”

The first theme that was evaluated in the qualitative study was about the modules of the Kusamala Program. All participants explained that they believed the 3 modules of the Kusamala Program were appropriate and that they learned from each of them. Several examples from participants also highlighted the interconnectedness between nutrition, WASH, and psychosocial stimulation modules.

The porridge should not be one type only. You can feed the child the one with groundnuts for a week and the other week we can mix soya, rice, beans to make porridge flour and this is important because it contains what the child's body requires. —Participant 6, IDI

It's good because you don't experience sudden diseases at home. Because if you have poor hygiene, many diseases arise at your home and children suffer from diarrhea and vomiting. You also need to dig a pit for waste disposal, and always have your house clean, cover all drinking water or boil before drinking. And find the child their own plate and keep it free from houseflies and after washing the plate, wipe it and keep it safe and when it's time for food just collect and put the food there. This helps the child grow with no problems. —Participant 15, FGD 3

The condition of my child was not good, and I didn't know that we can still be playing or talking to the child. I was thinking that since she was a patient, I was supposed to just leave her without playing with her. After I joined Kusamala at Moyo it's when I was told that I can still be talking to my child. —Participant 1, IDI

Additional modules that were suggested by 3 participants included teaching about infectious diseases like malaria and HIV. The 2 participants who suggested malaria as an important topic explained that it can be linked to the nutrition module. One participant also explained that program content could be designed to improve caregiver nutritional status in addition to child outcomes.

#### Duration and setting of the intervention: “To me it was not enough”

Seven participants in the qualitative study expressed that they would like the duration of the Kusamala Program to be longer, with one stating that the intervention should be across 7 d and the other 6 specifying that the duration of each session was too short.

To me it was not enough because I attended the lessons for few days then I was discharged from the hospital. —Participant 19, FGD 4

We learn for about an hour. It would be better if the time was extended to two hours. —Participant 3, FGD 1

A related subtheme that was underscored by 5 caregivers was that there were often interruptions, which was an additional reason according to some participants that the sessions should be longer.

Sometimes we end up not attending the whole session because at times we have to receive medication for the child and tend to the child, leaving the lesson in progress. —Participant 2, FGD 1

There are a lot of people in the ward. For instance, a doctor is making ward round, or it is time to get medicine; this makes one to lose concentration. —Participant 11, IDI

Six participants described other settings where they or their children received medical care or monitoring, such as health centers, postnatal clinics, or under-5 clinics, as possible settings for the Kusamala Program. Other hospital wards were described by 2 caregivers as possible opportunities for teaching other caregivers the messages from the program. A subtheme was identified, with the importance of prevention of malnutrition in children in the hospital or community recognized by 4 different caregivers.

You can also go to other wards where children have been admitted with other problems but not malnutrition, explaining to them because the child can be suffering from malaria but because of that, they can develop malnutrition. —Participant 6, IDI

#### Applying practices at home: “I practiced this at home until my child started walking”

All caregivers explained that they attempted to implement what they learned in the Kusamala Program when going home with specific examples.

I remember how I was encouraged. Because my child had stopped walking, but I was encouraged that I should help him stand, teach him, sometimes throw the ball to him and he will have a desire to walk. So, after discharge I practiced this at home until my child started walking. —Participant 16, FGD 3

Initially, I used to prepare the porridge and add a little oil. After adding the oil when the porridge is ready, I gave it to the child. What I learnt is that you can find vegetable soup, or sometimes an egg, add and mix then give the child. —Participant 11, IDI

Previously, I was not washing hands after changing the child's diapers; we were just washing hands before eating. But now, we wash hands after changing the child's diapers and before eating. —Participant 7, IDI

There were 2 instances in which caregivers said that they have significant difficulty in affording what they need to take care of their children. However, they both expressed that they still try to allocate money for caring for their children, with 1 caregiver explaining that it is less expensive than when a child must go to the hospital.

I try my best because I even made porridge flour from soya, beans and rice. When the flour is about to finish, I have to know how I can source money, or I can prepare porridge using maize flour and groundnut flour. The money one can spend when a child is admitted at the hospital is more than you can spend by just taking good care of the child. —Participant 6, IDI

The use of behavior change materials from the Kusamala Program was a subtheme that 4 caregivers discussed in the context of applying practices at home. Specifically, 2 mentioned the certificates that they received upon completing the Kusamala Program and 2 discussed the take-home images with nutrition and WASH messages.

When going home, I took a paper where pictures of foods and how to feed children were written. I showed other parents and they started also feeding their children properly. —Participant 13, IDI

Six caregivers also expressed that they shared messages that they learned from the Kusamala Program with family and other community members.

People in the village, I told them that during the admission at the hospital I was enrolled in Kusamala where I was taught about nutrition, thus feeding my child six food groups. I felt good that I had shared something important which would improve their lives and prevent them from being admitted to the hospital like me. —Participant 3, FGD 1

#### Overall sentiments about the Kusamala Program: “The lessons learned were an eye-opener”

Each of the 20 participants had positive sentiments about the Kusamala Program according to the qualitative study, and none had overall negative feelings towards it.

Despite the medical care in the hospital, Kusamala is important because the lessons are important and useful when we get back home. —Participant 3, FGD 1

The lessons learned were an eye-opener to problems arising due to poor nutrition and unhygienic conditions. —Participant 12, IDI

This program is good for the child because if the one responsible for the child is taught how they can feed the child and they follow it; they can be able to save a child whose life was at risk. —Participant 1, IDI

It was good in the sense that we learned a lot, especially on how to take care of our children. We used to prioritize other issues at the expense of our children. —Participant 18, FGD 4

## Discussion

This was the first trial to assess an NRU-based counseling program of multiple modules including a combination of nutrition and feeding, WASH, and psychosocial stimulation for caregivers of children with SAM admitted for inpatient treatment. We did not observe differences in developmental or nutritional outcomes in this cluster-randomized controlled trial between the intervention arm, which included primary caregivers and children involved in the Kusamala Program, and the comparison arm.

Our trial was not able to reach the previously calculated sample size due to a decline in NRU admissions, yet there was a low probability of a significant result if the trial was continued to achieve the target sample size based on the futility analysis. Specifically, MDAT composite *z*-scores and nutritional outcomes like WAZ were similar across arms in children with SAM 6 mo following discharge from inpatient treatment. The data also did not indicate improvements in HOME Inventory or dietary diversity scores. It is important to note that, due to the futility analysis, there may not be sufficient power to detect differences for these and other secondary outcomes.

The 2003 WHO guideline for inpatient management of SAM, which suggested that psychosocial stimulation interventions and nutritional counseling be delivered, do not indicate how best to implement these programs (8). The Kusamala Program differed from previous interventions in that it also incorporated nutrition and WASH modules and was designed to be more feasible to implement in the NRU settings.

We assessed the overall delivery of the Kusamala Program with fidelity assessments, which showed that delivery of the intervention by nurses in the NRU was of high quality. A majority of key messages were covered, behavior change materials given to participants, and counseling skills outlined in the Care for Child Development guide applied (22). In practice, intervention sessions were shorter than the planned 75 min, averaging almost 10 min below this target. Originally, the Kusamala Program was intended to be 90 min long, but was condensed based on results and experiences from a feasibility study of the intervention (20). Many caregivers felt that the program could be increased in duration, so involving additional NRU staff members in play sessions following counseling could potentially make it more feasible to extend the duration of sessions. The qualitative study also reflected that the Kusamala Program was viewed positively by caregivers involved in the program, with many participants outlining concepts they learned and aimed to apply at home.

Another important consideration with regard to the implementation is participant attendance, which was high on the first day of the program and gradually decreased over the course of the 4 d. The decline in attendance was predominantly due to children being discharged before the completion of the program. Children and caregivers were recruited within 3 d of inpatient admission per the trial protocol. Based on previous data of duration of stay, it was expected that most participants would remain in the NRU for the entirety of the intervention. The median duration of stay of surviving children in this trial was 7 (IQR: 5–9) d, and therefore it could be more appropriate for the intervention to occur as early as possible during the admission period.

However, results from this trial indicate that an inpatient-focused program of this intensity and duration is not sufficient to address long-lasting problems such as poor developmental outcomes in children admitted for SAM with complications. Although most children who survived 6 mo after discharge no longer had wasting, HAZs and WAZs remained low, with 53.0% of children stunted and 27.8% underweight, respectively, across both study arms. The outpatient mortality rate following treatment at the NRU was 14.7% and the readmission rate was 18.3% in children who survived initial inpatient treatment. These results highlight the nutritional and medical vulnerability of this patient population, which persists following discharge from the NRU.

Other previous studies of psychosocial stimulation interventions for children with SAM, which did show positive effects on child development, were rigorous during the inpatient period and continued beyond discharge from hospital (13–16). There are also more intensive caregiving programs that could be used, such as Reach Up, which involves visiting caregivers’ homes weekly to share and demonstrate how to use play materials with their children over a 2-y period (40). It may be worth evaluating extended versions of the Kusamala Program or other similar interventions with sessions beyond the inpatient period to have sustained effects on care practices.

Other approaches to program delivery could also be considered, such as mHealth as a means of behavior change communication or interventions that include conditional cash transfers to incentivize attending sessions in community settings. We also recommend having further community engagement when designing and implementing new interventions. This may be of similar value to the qualitative findings of our study, which highlighted potential areas of focus such as caregiver nutrition and illnesses like HIV and malaria. Furthermore, many participants in the qualitative study described sharing what they learned with their peers in the community and suggested that this type of program could be delivered in other hospital and community settings. While the Kusamala Program was designed for children with SAM admitted to NRUs, it could be adapted for other children and evaluated using similar mixed-methods approaches.

A limitation of this research was that many of the co-authors were involved in the design, implementation, and evaluation of the Kusamala Program, but we aimed to be as objective as possible and complete a critical appraisal using a mixed-methods approach. An additional limitation was that it was not possible to conduct subgroup analysis due to the small sample size, such as by child HIV status or age category, which could give insight into subgroups that could potentially benefit more from this type of intervention. The age range of children in this trial was wide, and this could lead to greater variance in MDAT *z*-scores and nutritional outcomes. These variables were included in analyses as covariates in the respective models. Furthermore, there was a high loss to follow-up, in part due to the COVID-19 pandemic causing home visits to be unsafe for study personnel and participants, which was accounted for as best as possible using multiple imputation. There were also many children who died during the study and could not be included in the main analyses, which is unfortunately common in children with SAM who require inpatient treatment.

Because of the complexities of research in this study population and setting, adaptive trial designs, which have been gaining popularity in recent years (41, 42), could be considered and should be planned a priori whenever possible. In our trial, futility analysis was completed to decide whether to continue or to halt the study and avoid unnecessarily utilizing resources and involving participants in data collection. The futility analysis meant that it was possible to be underpowered to conduct a range of secondary analyses. This analysis also introduces an additional source of bias, so this should be considered by any investigators conducting meta-analysis using these data (43, 44).

In conclusion, this mixed-methods cluster-randomized controlled trial did not support the effectiveness of the Kusamala Program to improve developmental or nutritional outcomes in children with SAM 6 mo after NRU discharge. However, the quality of delivery of the Kusamala Program in the NRU was high based on the fidelity assessments, and qualitative results showed that this intervention was viewed positively by caregivers. This study has highlighted the urgency to establish and implement enhanced programs for primary caregivers and children with SAM that begin at admission to NRUs but continue beyond the inpatient period in community settings.

## Data Availability

Data have been made available in a repository here: https://doi.org/10.5683/SP2/F8SJBV.

## Notes

This research was supported by Grand Challenges Canada. Grand Challenges Canada is funded by the Government of Canada and is dedicated to supporting Bold Ideas with Big Impact®. Trainee support for this project was awarded from the Canadian Institutes of Health Research.

Author disclosures: The authors report no conflicts of interest. The sources of support had no role in designing this study, carrying out study activities or analysis, or writing the manuscript.

Abbreviations used: COVID-19, coronavirus disease 2019; FGD, focus group discussion; HAZ, height-for-age *z*-score; HOME, Home Observation of the Measurement of the Environment; IDI, in-depth interview; MDAT, Malawi Developmental Assessment Tool; MUAC, midupper arm circumference; NRU, nutritional rehabilitation unit; SAM, severe acute malnutrition; WASH, water, sanitation, and hygiene; WAZ, weight-for-age *z*-score; WHZ, weight-for-height *z*-score.
